# Analysis of prostate intensity‐ and volumetric‐modulated arc radiation therapy planning quality with PlanIQ^TM^

**DOI:** 10.1002/acm2.13233

**Published:** 2021-03-25

**Authors:** Motoharu Sasaki, Yuji Nakaguuchi, Takeshi Kamomae, Akira Tsuzuki, Satoshi Kobuchi, Kenmei Kuwahara, Shoji Ueda, Yuto Endo, Hitoshi Ikushima

**Affiliations:** ^1^ Institute of Biomedical Sciences Tokushima University Graduate School Tokushima Japan; ^2^ Toyo Medic Co.,Ltd Tokyo Japan; ^3^ Department of Radiology Nagoya University Graduate School of Medicine Nagoya Aichi Japan; ^4^ Department of Radiological Technology Kochi University Hospital Kochi Japan; ^5^ Graduate School of Health Sciences Tokushima University Tokushima Japan; ^6^ School of Health Sciences Tokushima University Tokushima Japan

**Keywords:** Feasibility DVH^TM^, IMRT, PlanIQ^TM^, Prostate cancer, VMAT

## Abstract

**Purpose:**

The purpose of this study was to assess the quality of treatment planning using the PlanIQ^TM^ software and to investigate whether it is possible to improve the quality of treatment planning using the “Feasibility dose‐volume histogram (DVH)^TM^” implemented in the PlanIQ^TM^ software.

**Methods:**

Using the PlanIQ^TM^ software, we retrospectively analyzed the learning curve regarding the quality of the treatment plans for 148 patients of prostate intensity‐modulated radiation therapy and volumetric‐modulated radiation therapy performed at our institution over the past eight years. We also sought to examine the possibility of improving treatment planning quality by re‐planning in 47 patients where the quality of the target dose and the dose limits for organs at risk (OARs) were inadequate. The re‐planning treatment plans referred to the Feasibility DVH^TM^ implemented in the PlanIQ^TM^ software and modified the treatment planning system based on the target dose and OAR constraints.

**Results:**

Analysis of the learning curve of the treatment plans quality using PlanIQ^TM^ software retrospectively showed a trend of improvement in the treatment plan quality from year to year. The improvement in the treatment plans quality was more influenced by dose reduction in the OARs than by target coverage.

In all cases where re‐planning was performed, the improvement in the treatment plan's quality resulted in a better treatment plan than the one adopted for delivery to patients in the clinical plan.

**Conclusions:**

The PlanIQ^TM^ provided insights into the quality of the treatment plans at our institution and identified problems and areas for improvement in the treatment plans, allowing for the development of appropriate treatment plans for specific patients.

## 1 INTRODUCTION

In recent years, the usage rate of intensity‐modulated radiation therapy (IMRT) and volumetric‐modulated radiation therapy (VMAT) has increased across institutions, worldwide. These treatments allow for focused dose delivery to the target and reductions in the dose to the organs at risk (OARs).^1^, ^2^ Intensity‐modulated radiation therapy and VMAT are routinely performed using dose constraint sheets for the guidance of the plans determined by each institution. However, dose constraint sheets do not provide explicit information on the quality of planning that can be optimally achieved for each patient.^3^, ^4^, ^5^ Instead, they contain recommendations pertaining to OAR dose limits. Therefore, satisfaction of the dose constraint sheet alone is insufficient in the determination of whether the treatment plan being developed for a particular patient is appropriate. Typically, the treatment planner indicates the target dose and OAR constraints as inputs. Optimizers are programmed to identify a minimum cost function that incorporates the target dose and OAR dose constraints required for the treatment plan entered by the planner.^6^, ^7^

**Fig. 1:**
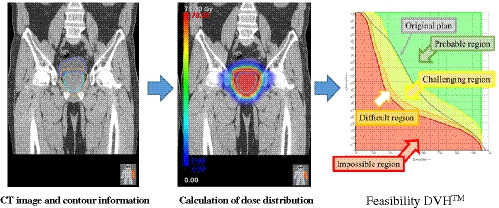
Feasibility DVH^TM^ calculation method. Computed tomography images, contour information, and prescription dose for the target are defined, and the ideal dose distribution is calculated. Then, based on the calculated dose distribution and contour information, the feasible dose volume histogram (DVH) is calculated.

In recent years, PlanIQ^TM^ (Sun Nuclear, Melbourne, Florida, USA) has been marketed as a software for the analysis of treatment plan quality metrics. It uses a Feasibility dose‐volume histogram (DVH)^TM^, which is based on a falloff of the ideal dose from the prescribed dose at the target boundary, allowing for the quantitative determination of impossible regions (red), difficult regions (orange), challenging regions (yellow), and probable regions (green) (Fig. 1). “Impossible DVH (red)” is defined as the DVH generated using the minimum dose that an off‐target voxel must receive given 100% target coverage. Studies that used the PlanIQ^TM^ software have reported improvements in the treatment plan quality.^8^, ^9^, ^10^, ^11^, ^12^, ^13^ Recently, PlanIQ^TM^ was integrated into Autoplan®, which is implemented in Pinnacle and has been clinically applied.^9^, ^10^ Perumal et al.^9^ compared the dosimetry results of optimization using Autoplan® and treatment planning based on OAR targets obtained from PlanIQ^TM^ in five patients with different disease sites. They reported that when the clinical targets suggested by PlanIQ^TM^ were used for Autoplan®‐based optimization, the quality of the plan was significantly improved without the use of many iterative steps. They also noted that the use of PlanIQ^TM^ was useful as it allowed for the obtainment of information on how the OAR dose can be reduced without compromising the target coverage before optimization. The authors of that study^9^ also concluded that the planners were able to define clinical targets tailored to each patient's anatomy in advance, leading to significant reductions in the OAR dose. Xia et al.^10^ reported about the use of VMAT in 10 lung cancer patients. They compared the value of clinically accepted manual planning with Autoplan®, based on the PlanIQ^TM^ Feasibility DVH^TM^ and reported that Autoplan®‐customized treatment plans for specific patients, proposed based on the PlanIQ^TM^ Feasibility DVH^TM^, resulted in better dose reductions to the lungs and were useful in improving the plan's quality. There are currently no reports on the quality of treatment plans retrospectively analyzed at a single center and the accuracy of DVH provided by the Feasibility DVH^TM^.

In this study, we aimed to retrospectively analyze the learning curve for treatment plan quality for prostate IMRT and VMAT performed at our institution over the past eight years. The PlanIQ^TM^ software was used to assess the quality of treatment planning. As per the learning curve analysis, if the quality of the treatment plan improves yearly, the clinical outcomes too are likely to improve. If the treatment plan’s quality is stagnant or worsens with each year, patients’ clinical outcomes may not improve unless the method of treatment planning is reviewed. We retrospectively analyzed the treatment plans previously used at our institution to determine their quality. We believe that gaining an understanding of the quality of the treatment plans used at our institution can aid in the identification of problems and areas for improvement, enabling the development of appropriate treatment plans for specific patients.

We also sought to examine the possibility of improving the quality of treatment planning by re‐planning in cases where the quality of the target dose and OAR dose limit were inadequate. The re‐planning treatment plan referred to the Feasibility DVH^TM^ implemented in the PlanIQ^TM^ software and modified the treatment planning system (TPS) on the basis of the target dose and OAR constraints. We assessed whether the re‐planning treatment plan could reproduce the DVH provided by the Feasibility DVH^TM^, as it may prove useful in IMRT and VMAT treatment planning.

## 2 METHODS

### 2.A Patients and clinical plans

This study included data of 148 patients who underwent prostate sliding window IMRT and VMAT during the eight years from 2012 to 2019. Table 1 shows the number of patients who underwent IMRT and VMAT each year. The linear accelerator used for radiotherapy was NovalisTx (Varian Medical Systems, Palo Alto, CA, USA) and the energy value employed was 15 MV‐X. The TPS used was Eclipse (Varian Medical Systems, Palo Alto, CA, USA) versions 8.9.17 and 11.0.31, and the dose calculation algorithm used was anisotropic analytical algorithm. The VMAT optimization algorithm was the progressive resolution optimizer algorithm 3 (PRO3) from version 10 onwards; PRO2 was used prior to that. Sliding window IMRT with 7 gantry angles (0°, 55°, 105°, 155°, 205°, 255°, and 305°) was used. The VMAT was performed using two full gantry arcs. All treatment plans used a dose calculation grid size of 2.5 mm × 2.5 mm × 2.5 mm. From 2012 to 2014, treatment planning was performed using both IMRT and VMAT for all cases, and the treatment plan with higher efficacy was selected. From 2015 onwards, with the upgrading of TPS, the calculation time of one treatment plan in VMAT planning was significantly reduced. As a result, multiple VMAT plans could be developed, and only treatment plans for VMAT were implemented.

**Table 1:** Number of IMRT and VMAT cases from 2012 to 2019.

	IMRT	VMAT	Total
2012	6	11	17
2013	18	7	25
2014	7	13	20
2015	0	19	19
2016	0	11	11
2017	0	15	15
2018	0	21	21
2019	0	20	20

IMRT: intensity‐modulated radiation therapy, VMAT: volumetric‐modulated radiation therapy.

The contour data used for treatment planning were: clinical target volume (CTV) and planning target volume (PTV) excluding the rectum and rectum and bladder. PTV excluding the rectum contour was used for both optimization and dose evaluation. A radiation oncologist defined all the contours according to our institution’s contouring protocol.^1^ The CTV was defined as the prostate volume plus a portion of the seminal vesicle located within 2 cm of the prostate. Per Radiation Therapy Oncology Group guidelines,^14^ the rectum volume is defined as the area between the sciatic and descending colon and rectum, or up to 15 cm. However, in the present study, rectum contour was defined as six slices superior and inferior to the CTV, minimizing differences in the rectal contour. The margin from the CTV to PTV was 8 mm, excluding the dorsum, and 6 mm for the dorsum only. Our prescribed dose was 78 Gy in 39 fractions, including 95% of the PTV excluding the rectum (D95%).

### 2.B Change in the learning curve of the treatment plan

In this study, the quality of the treatment plan was assessed using a scoring mechanism called the “Plan Quality Metric (PQM),” implemented in PlanIQ™ proposed by Nelms^11^ setting. The relative importance of each item can be defined by a score within the target dose and the OAR dose limits. Scoring the entire treatment plan based on prioritization can serve as an objective assessment of the treatment plan and a benchmark for the achievement of continuous improvement.

The PQM scoring table created in this study is shown in Table 2. It was determined by accounting for the dose constraints of our institution^1^ and previous treatment outcomes associated with prostate IMRT and VMAT. The PQM scoring table investigated in this study comprised nine subcomponents. For each subcomponent, a score was calculated based on a unique metric amount and sub‐metric. The nine subcomponents were created with three target coverage and six OAR dose limits. The values between the maximum and minimum scores in the PQM scoring table were linearly interpolated. The setting of each PQM metric maximum and minimum was determined by the mean ± two standard deviations from the dose constraint sheets of the 148 patients retrospectively analyzed in this study. As an example of the evaluation of target concentration, PTV excluding the rectum is described below. PTV excluding the rectum was evaluated at D98% and D2%, where 0 and 25 points were assigned at 75.8 and 77.3 Gy, respectively, since D98% is an indicator of the lowest dose, and the higher the dose, the higher the score. In contrast, for D2%, the score was 0 at 84.9 Gy and 25 at 81.9 Gy, and the lower the dose, the higher the score, because D2% is an indicator of the maximum dose. However, only V100% of the CTV was set as 100%, as the maximum value exceeded 100% when two standard deviations were added to the mean value. Next, for OAR, since the dose should be minimized, we evaluated the percentage of volume occupied by the high‐dose region in dose distribution. For example, for the urinary bladder, a score of 0 was assigned for 25% of the V65 Gy, and a score of 10 for 5.3% of the V65 Gy, and a higher score was assigned for a smaller percentage of the volume in the evaluated dose area. Other evaluation indices for OAR were set in the same way. The table was reviewed and approved by radiation oncologists after a discussion and defined by a team of four expert planners for the determination of its relative value. Therefore, we believe that there is no ambiguity pertaining to the importance of each of the PQM scoring tables in terms of their relative scores, as they reflect the treatment plan’s policies and objectives.

**Table 2:** PQM scoring table.

Structure	Metric	Minimum		Maximum	
		Criteria	Score	Criteria	Score
CTV	V100% (%)	99.1 (%)	0	100.0 (%)	20
PTV excluding the rectum	D98% (Gy)	75.8 (Gy)	0	77.3 (Gy)	25
PTV excluding the rectum	D2% (Gy)	84.9 (Gy)	0	81.9 (Gy)	25
Rectum	V75 Gy (%)	5.4 (%)	0	1.2 (%)	30
Rectum	V70 Gy (%)	11.9 (%)	0	5.7 (%)	30
Rectum	V60 Gy (%)	21.9 (%)	0	13.1 (%)	30
Rectum	V40 Gy (%)	42.9 (%)	0	28.0 (%)	20
Bladder	V65 Gy (%)	25.0 (%)	0	5.3 (%)	10
Bladder	V40 Gy (%)	47.8 (%)	0	17.6 (%)	10

CTV: Clinical target volume, PTV: planning target volume; PQM: plan quality metric.

Additionally, the Feasibility DVH^TM^ implemented in PlanIQ^TM^ software, defines the ideal dose distribution using CT images, contour information, and a given dose to the target (Fig. 1). The PQM score, called the “Adjusted Planning Quality Metric (APQM),” was calculated on the basis of the ideal treatment was predicted plan based on CT images and contour information. Since APQM is the calculation of PQM scores for an ideal treatment plan, the allocation of points is identical to the allocation shown in Table 2.

First, we assessed the quality and validity of the clinical treatment plan used in this study. We assessed the correlation between the overall score of the clinical treatment plan (PQM total score) and the overall ideal treatment plan score, as calculated by the Feasibility DVH^TM^ (APQM total score).

Next, we assessed the learning curve of treatment plan quality for each year from 2012 to 2019, which was evaluated as the cumulative frequency ratio by the total PQM score of the treatment plans adopted in the clinical plan. The nine subcomponents of the PQM scoring table were also assessed for changes in the learning curve effect associated with the treatment plan for each year from 2012 to 2019. Moreover the nine subcomponents of the PQM scoring table and the PQM total scores for each year of the treatment plan from 2012 to 2019 were evaluated using the Mann–Whitney U significance test, which is a two‐group unpaired significance test.

### 2.C Potential for treatment plan quality improvement

**Fig. 2:**
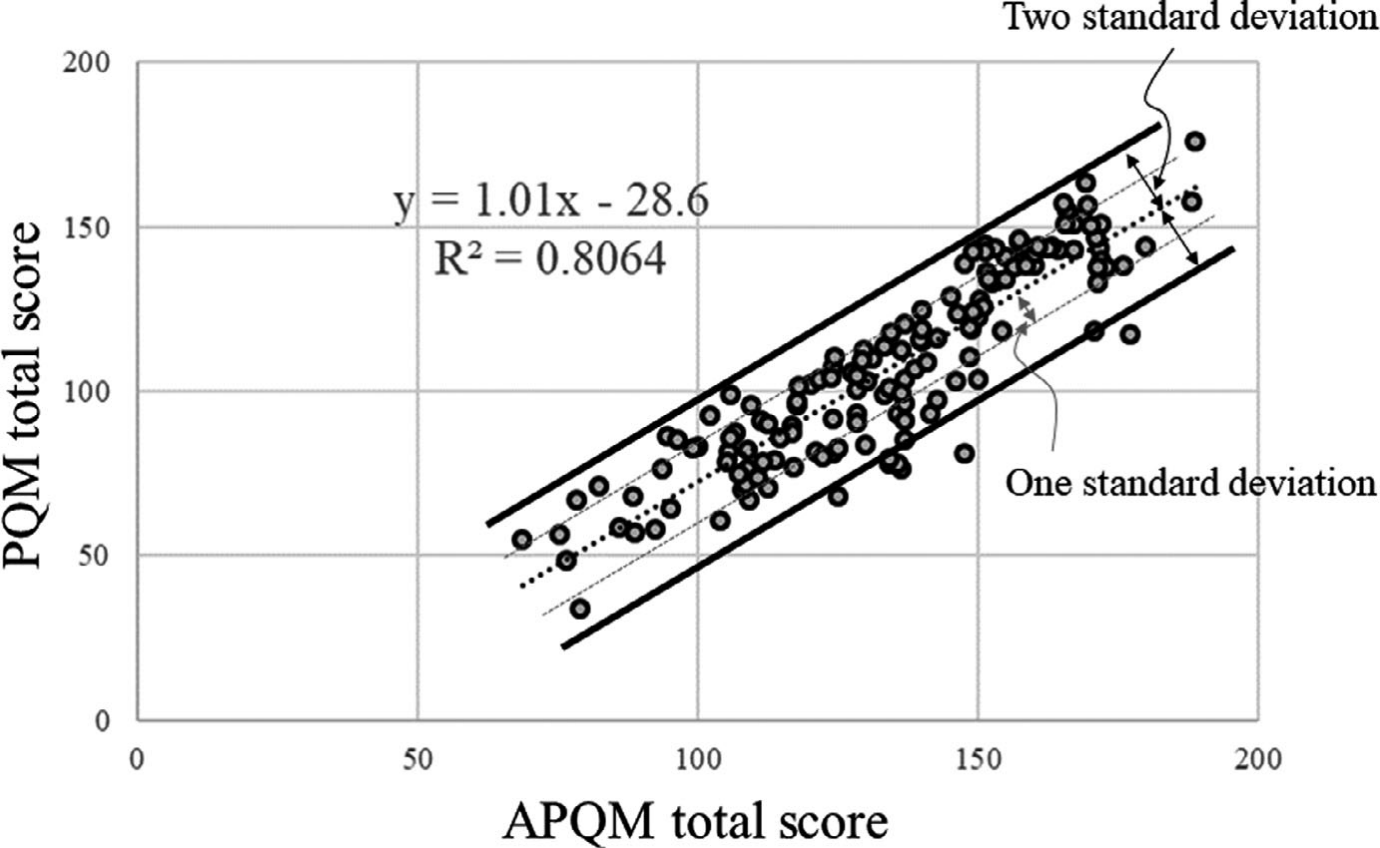
Correlation between the APQM total score and PQM total score of 148 patients underwent prostate IMRT and VMAT at our institution from 2012 to 2019. The black dotted line indicates a linear approximation. The solid black line shows two standard deviations from the linear approximation, and the dotted gray line shows one standard deviation from the linear approximation. APQM: adjusted plan quality metric, PQM: plan quality metric.

The re‐treatment plan for 47 patients was evaluated for the investigation of whether the treatment plan’s quality could be improved with reference to the Feasibility DVH^TM^ (Fig. 2). A breakdown of the number of IMRT and VMAT re‐treatment plans by year of the original treatment plan is shown in Table 3. We used the “difficult region (orange)” of the Feasibility DVH^TM^ as a reference point for our re‐treatment planning, which, in our experience, does not compromise the target coverage degree or OAR dose. The re‐treatment plan was implemented in version 11.0.31 with two arc VMAT. The assessment compared the PQM total score, which is the overall score of the treatment plan for patient delivery in the clinical plan, with the re‐planned PQM (R‐PQM) total score, the overall score of the re‐treatment plan. We also compared the APQM total score with the R‐PQM total score.

**Table 3:** Breakdown of the number of IMRT and VMAT retreatment plans by year of the original treatment plan.

	IMRT	VMAT	Total
2012	3	4	7
2013	8	7	15
2014	2	5	7
2015	0	6	6
2016	0	4	4
2017	0	1	1
2018	0	5	5
2019	0	2	2

IMRT: intensity‐modulated radiation therapy, VMAT: volumetric‐modulated radiation therapy.

## 3 RESULTS

### 3.A Change in the treatment plan learning curve

Figure 2 shows the correlation between the APQM total score and PQM total score in the 148 patients who underwent prostate sliding window IMRT and VMAT at our institution from 2012 to 2019. The R^2^ was 0.8064, showing a strong correlation.

**Fig. 3:**
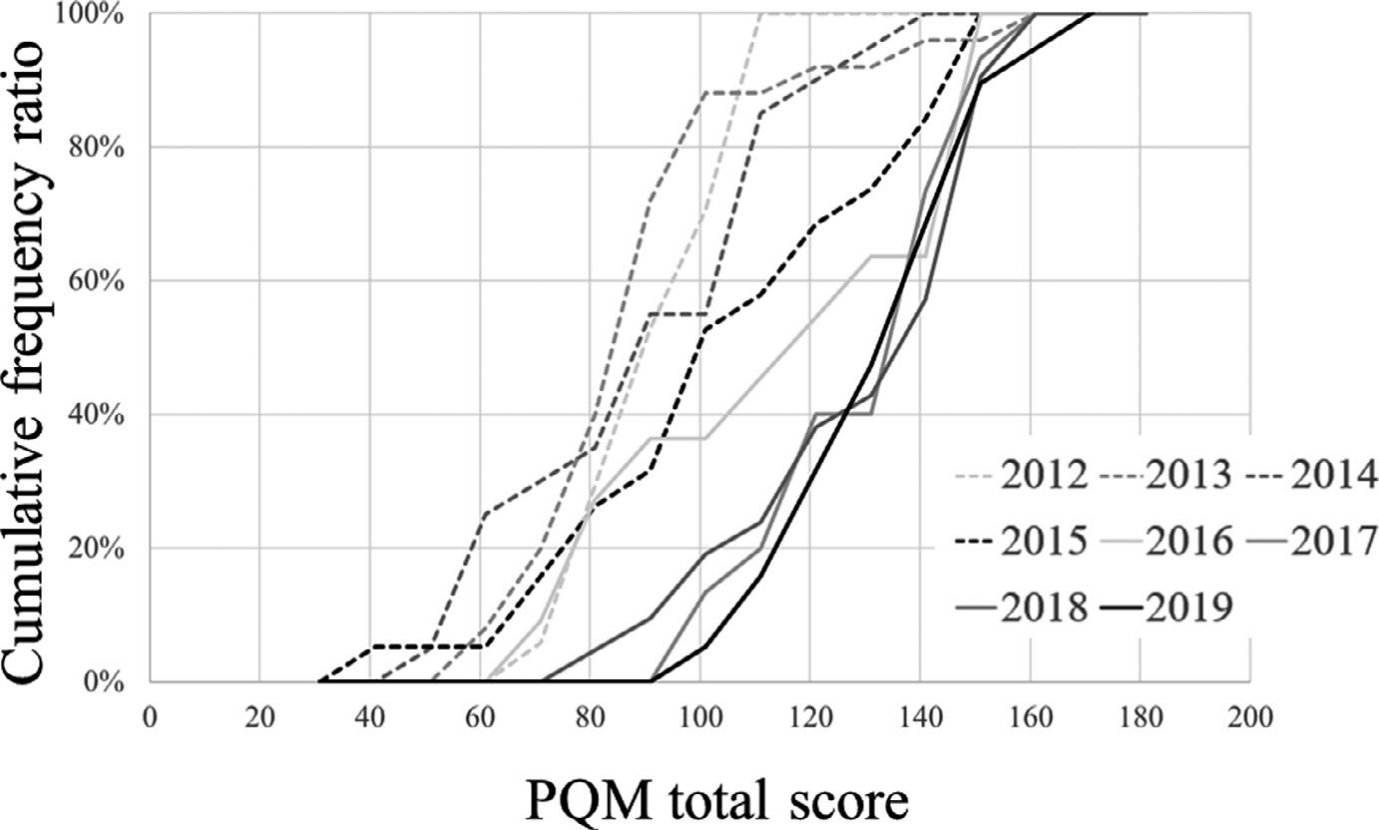
Cumulative frequency ratios by PQM total score for the treatment plans were adopted by the clinical plans for each year from 2012 to 2019. The cumulative frequency distribution indicates the cumulative percentage of PQM scores for each year's treatment plan. For example, a cumulative frequency distribution of 0% indicates the treatment plan with the lowest PQM total score of the treatment plan for that year; a cumulative frequency distribution of 50% indicates the treatment plan with the median PQM total score of the treatment plan for that year; and a cumulative frequency distribution of 100% indicates the treatment plan with the highest PQM total score of the treatment plan for that year. Therefore, the cumulative frequency distribution shows that the right side of the graph indicates that the quality of the treatment plan is better. PQM: plan quality metric.

Figure 3 shows the cumulative frequency ratios by the PQM total score for the clinical treatment plans adopted in each year from 2012 to 2019. The Eclipse version used from 2012 to 2014 was 8.9.17 and 11.031 after that. The PQM total score for the 50% cumulative frequency ratio from 2012 to 2014 ranged between 84.13 and 89.75. The PQM total score for the 50% cumulative frequency ratio from 2015 to 2019 ranged between 99.75 and 136.00, showing an improving trend over the years; from 2017 onwards, an even more substantial trend of improvement was observed over time.

The significant differences in the PQM total scores from 2012 to 2019 with the respective annual PQM total scores are shown in Table 4. The PQM total scores for 2012, 2014, and 2015 showed significant differences in each year compared with the PQM total scores from 2017 to 2019. The PQM total scores for 2013 showed significant differences from the PQM total scores of 2016–2019, and no significant differences between the PQM total scores from 2017 to 2019 were observed for each year.

**Table 4:** Results of Mann–Whitney *U* significance test, a two‐group unpaired significance test, for the nine subcomponents of the PQM scoring table and the PQM score total for each year of the treatment plan from 2012 to 2019. Items with statistically significant differences are in bold and underlined.

	2012	2013	2014	2015	2016	2017	2018	2019
CTV V100%								
2012	x	0.187	0.940	0.731	**0.002**	**<0.001**	**0.005**	**0.013**
2013	x	x	0.205	0.389	**0.041**	**0.016**	0.140	0.330
2014	x	x	x	0.857	**0.003**	**0.001**	**0.017**	0.063
2015	x	x	x	x	**0.018**	**0.007**	0.069	0.141
2016	x	x	x	x	x	0.610	0.123	**0.036**
2017	x	x	x	x	x	x	0.252	**0.017**
2018	x	x	x	x	x	x	x	0.297
2019	x	x	x	x	x	x	x	x
PTV excluding rectum D98%								
2012	x	**0.005**	**0.026**	0.639	0.134	**0.024**	0.885	0.220
2013	x	x	0.385	**0.001**	**<0.001**	**<0.001**	**0.002**	**<0.001**
2014	x	x	x	**0.010**	**0.002**	**<0.001**	**0.018**	**0.001**
2015	x	x	x	x	0.145	0.083	0.810	0.411
2016	x	x	x	x	x	0.760	0.123	0.451
2017	x	x	x	x	x	x	**0.046**	0.283
2018	x	x	x	x	x	x	x	0.297
2019	x	x	x	x	x	x	x	x
PTV excluding rectum D2%								
2012	x	0.155	0.104	0.379	0.926	0.390	0.367	**0.024**
2013	x	x	0.891	0.427	0.324	0.346	0.316	0.326
2014	x	x	x	0.627	0.183	0.330	0.514	0.369
2015	x	x	x	x	0.420	0.973	0.915	0.149
2016	x	x	x	x	x	0.721	0.506	0.054
2017	x	x	x	x	x	x	0.975	**0.043**
2018	x	x	x	x	x	x	x	0.151
2019	x	x	x	x	x	x	x	x
Rectum V75 Gy								
2012	x	0.838	0.158	0.684	**0.019**	**0.006**	**<0.001**	**<0.001**
2013	x	x	0.157	0.610	**0.029**	**0.002**	**<0.001**	**<0.001**
2014	x	x	x	0.647	0.123	0.055	**0.001**	**0.001**
2015	x	x	x	x	0.216	**0.027**	**<0.001**	**0.009**
2016	x	x	x	x	x	0.281	0.123	0.476
2017	x	x	x	x	x	x	0.214	0.755
2018	x	x	x	x	x	x	x	0.348
2019	x	x	x	x	x	x	x	x
Rectum V70 Gy								
2012	x	0.599	0.270	0.121	0.066	**<0.001**	**<0.001**	**<0.001**
2013	x	x	0.599	0.387	0.324	**<0.001**	**<0.001**	**<0.001**
2014	x	x	x	0.380	0.157	**<0.001**	**<0.001**	**<0.001**
2015	x	x	x	x	0.471	**0.007**	**0.001**	**0.003**
2016	x	x	x	x	x	0.237	0.104	0.212
2017	x	x	x	x	x	x	0.465	0.987
2018	x	x	x	x	x	x	x	0.676
2019	x	x	x	x	x	x	x	x
Rectum V60 Gy								
2012	x	0.311	0.357	0.076	**0.025**	**<0.001**	**<0.001**	**<0.001**
2013	x	x	0.758	0.362	0.207	**<0.001**	**<0.001**	**<0.001**
2014	x	x	x	0.258	0.072	**<0.001**	**<0.001**	**<0.001**
2015	x	x	x	x	0.420	0.066	**0.015**	**0.002**
2016	x	x	x	x	x	0.413	0.457	0.104
2017	x	x	x	x	x	x	0.849	0.298
2018	x	x	x	x	x	x	x	0.251
2019	x	x	x	x	x	x	x	x
Rectum V40 Gy								
2012	x	0.131	0.125	0.138	**<0.001**	**<0.001**	**<0.001**	**<0.001**
2013	x	x	0.758	0.981	**0.022**	**0.001**	**0.003**	**<0.001**
2014	x	x	x	0.708	**0.012**	**<0.001**	**0.001**	**<0.001**
2015	x	x	x	x	**0.033**	**0.006**	**0.007**	**<0.001**
2016	x	x	x	x	x	0.799	1.000	0.060
2017	x	x	x	x	x	x	0.899	0.064
2018	x	x	x	x	x	x	x	**0.020**
2019	x	x	x	x	x	x	x	x
Bladder V65 Gy								
2012	x	0.898	0.167	0.093	0.853	**0.016**	**0.023**	**0.001**
2013	x	x	0.379	0.066	0.477	**0.008**	**0.022**	**0.003**
2014	x	x	x	**0.003**	0.095	**<0.001**	**0.001**	**<0.001**
2015	x	x	x	x	0.216	0.410	0.421	0.194
2016	x	x	x	x	x	0.054	0.088	0.016
2017	x	x	x	x	x	x	0.751	0.882
2018	x	x	x	x	x	x	x	0.865
2019	x	x	x	x	x	x	x	x
Bladder V40 Gy								
2012	x	0.939	0.390	0.129	0.487	0.109	0.073	0.133
2013	x	x	0.472	0.152	0.324	0.083	**0.044**	**0.028**
2014	x	x	x	**0.010**	0.060	**0.003**	**<0.001**	**<0.001**
2015	x	x	x	x	0.672	0.811	0.668	0.728
2016	x	x	x	x	x	0.330	0.208	0.227
2017	x	x	x	x	x	x	0.874	0.780
2018	x	x	x	x	x	x	x	0.442
2019	x	x	x	x	x	x	x	x
Total								
2012	x	0.254	0.845	0.165	0.082	**<0.001**	**<0.001**	**<0.001**
2013	x	x	0.568	0.056	**0.029**	**<0.001**	**<0.001**	**<0.001**
2014	x	x	x	0.175	0.054	**<0.001**	**<0.001**	**<0.001**
2015	x	x	x	x	0.611	**0.017**	**0.009**	**0.004**
2016	x	x	x	x	x	0.180	0.088	0.072
2017	x	x	x	x	x	x	0.751	0.564
2018	x	x	x	x	x	x	x	0.938
2019	x	x	x	x	x	x	x	x

PQM: plan quality metric.

**Fig. 4:**
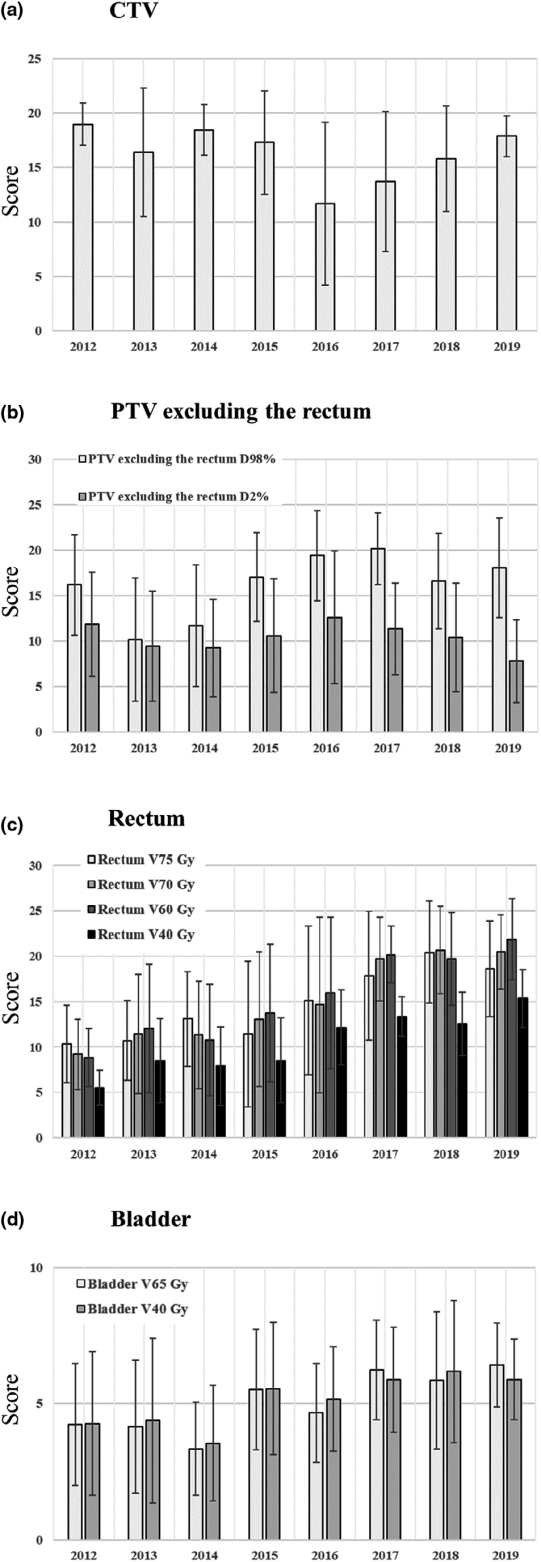
Results of three target coverage scores and six organs at risk dose constraints created in the PQM scoring table. (a) Clinical target volume; (b) planning target volume excluding the rectum; (c) rectum; (d) bladder. Error bars indicate one standard deviation. DX%: The dose value covers volume X, VX Gy: Percentage of the volume irradiated by X Gy.

Figures 4(a) and 4(b) show the V100% score of the CTV and D98% and D2% scores of the PTV excluding the rectum, which are the three target coverage areas created in the PQM scoring table. The significant differences between the sub‐metric scores from 2012 to 2019 and the sub‐metric scores of the respective years are shown in Table 4. The results shown in Table 4 shows that the V100% score of the CTV scores for 2012 showed significant differences in each year from the V100% score of the CTV scores from 2016 to 2019; the V100% score of the CTV for 2013 and 2015 showed significant differences from the V100% score of the CTV from 2016 to 2017; the V100% score of the CTV for 2014 showed significant differences from the V100% score of the CTV for 2016 through 2018; the V100% score of the CTV for 2016 and 2017 showed significant differences from the V100% score of the CTV for 2019. For PTV excluding the rectum, D2% showed significant differences between 2012 and 2019 and between 2017 and 2019; D98% showed more significant differences between the respective years than did D2%.

Figure 4(c) shows the V75 Gy, V70 Gy, V60 Gy, and V40 Gy for the rectum, and Fig. 4(d) the V65 Gy and V40 Gy scores for the bladder, which are the dose limits of the six OARs. The mean score for each subcomponent of the rectum tended to increase over the years [Fig. 4(c)]. Of the subcomponent mean scores of each rectal subcomponent, the V60 Gy score showed the greatest increase, followed by the V70 Gy score. The mean score of each subcomponent of the bladder showed a slight increasing tendency with every passing year [Fig. 3(e)]. Compared to the mean score for each subcomponent of the rectum, the mean score for each subcomponent of the bladder tended to increase to a lower degree. For OAR, more significant differences were observed in scores in the low‐dose region than in the high‐dose region.

### 3.B Potential for treatment plan quality improvement

**Fig. 5:**
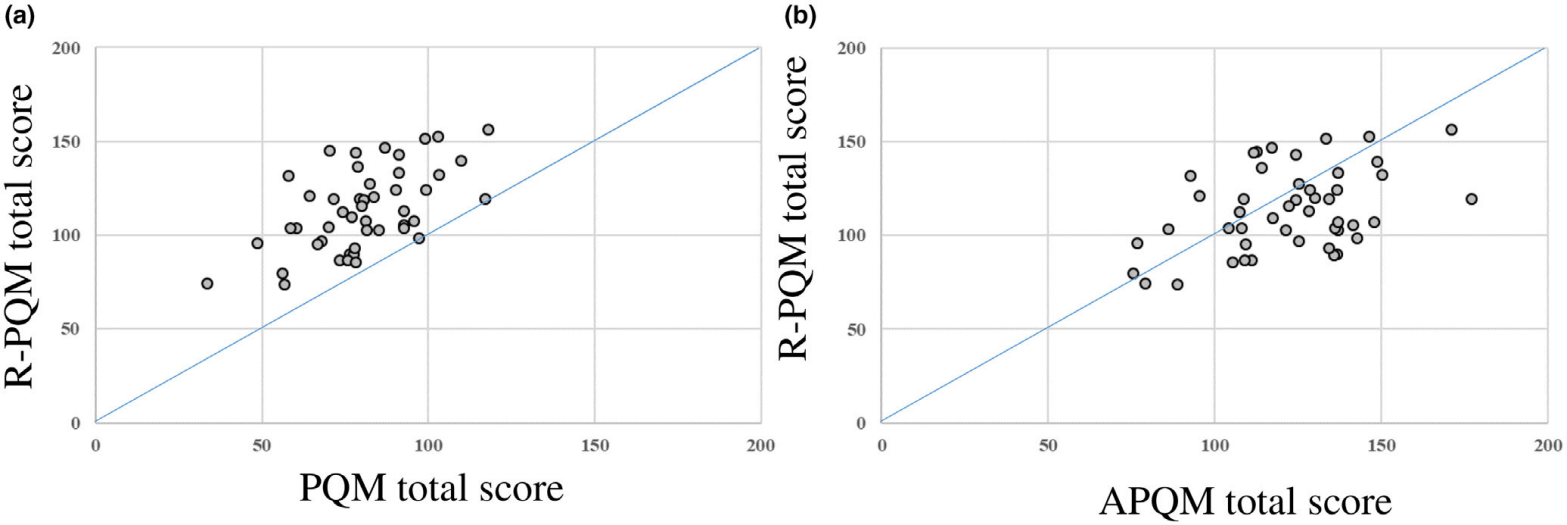
Results of re‐treatment plan in 47 patients. (a) PQM total score and R‐PQM total score, (b) PQM total score, and APQM total score. APQM: adjusted plan quality metric, PQM: plan quality metric, R‐PQM: re‐planned PQM.

In the re‐treatment planning, we used the “difficult region (orange)” of the Feasibility DVH^TM^" as a reference to set the optimization object. Figure 5(a) shows the results of the PQM total score (the overall score of the treatment plan adopted for patient delivery in the clinical plan) and the R‐PQM total score (the overall score of the re‐treatment plan). Figure 5(b) shows the results of the comparison performed between the APQM total score and R‐PQM total score, which is the overall score of the ideal treatment plan proposed by the Feasibility DVH^TM^. As shown in Fig. 5(a), the total score was higher than that for the treatment plan adopted for patient delivery in the clinical plan in all the cases in which re‐planning was performed. The R‐PQM total score was higher than the PQM total score, with the mean ± two standard deviations value of 33.19 ± 18.85. Figure 5(b) shows that the R‐PQM total score was higher in some treatment plans than the APQM total score in 13 of the 47 cases. The total re‐treatment plan score was lower than the total ideal treatment plan score proposed by the Feasibility DVH^TM^ by a mean ± two standard deviation value of −8.51 ± 23.63.

## 4 DISCUSSION

The present study retrospectively analyzed the IMRT and VMAT plans implemented over the past eight years using the PlanIQ^TM^ software for learning curve evaluation. The results in Fig. 3 show that the cumulative frequency percentage of the most recent treatment plan from 2017 to 2019 showed an improvement in the quality of the treatment plan (increase in the average PQM total score) and a decrease in variability (decrease in the difference between the minimum and maximum PQM total score) compared to the earlier treatment plans. This improvement is reflected in the data in Table 4 as well.

The concept of PQM implementation in the PlanIQ^TM^ software was created by Nelms et al.^11^ for the quantification of treatment plan quality variability. They showed that treatment planner ability is not statistically dependent on technical parameters (TPS, modality, and complexity of the plan),^11^ and also concluded that the considerable variation in the quality of treatment plans may be attributed to the planner’s general skill. Therefore, PlanIQ^TM^ does not necessarily improve the planners' skills but provides an estimate of what is clinically feasible and a template for optimization objectives. The PlanIQ^TM^ software used in this study is a tool that is useful in the assessment of consistency, quantifiability, and reproducibility; we believe that the retrospective investigation of the learning effects of treatment planning, as in this study, is essential in improving treatment outcomes in such settings. We also believe that the dissemination of awareness on the problems and areas for improvement associated with treatment planning at each institution can aid planners in improving their skills and minimize variations in planners’ skills at each facility. Ultimately, we believe that if the degree of variation in planners’ skills can be minimized, the average quality of the treatment provided in a facility can be improved.

We discussed the improvements observed in the PQM total score since 2015. The version of Eclipse used from 2012 to 2014 was 8.9.17, and the optimization algorithm was PRO2; the version of Eclipse used thereon was 11.0.31, and the optimization algorithm was PRO3. A comparison of the treatment plans between these two optimization algorithms has been previously peformed.^15^ Vanetti et al.^15^ found that PRO3 yielded better treatment planning results than PRO2. Similarly, we believe that the overall PQM score of the treatment plans in this study was better after 2015 than before 2014 due to differences in the optimization algorithm. We also believe that the further superiority of the PQM total score from 2017 to 2019 compared that from 2015 to 2016 based on the results of the Mann–Whitney U test shown in Table 4 is due to the fact that the optimization setting with PRO3 became more familiar and mature in the two years from 2015 to 2016. The value of the optimization algorithm of Eclipse, the TPS used in this study, has been reported in recent years, with some clinical studies using photon optimizer (PO) instead of PRO, with excellent results.^16^, ^17^, ^18^ Therefore, future analyses using the PO should be performed.

Based on the results shown in Table 4, the minimum value of the criterion is 99.1% and the maximum value is 100% for V100% of CTV, which is quite a narrow range; thus, we believe that the results show a significant difference albeit the small difference. We believe that the dip in the V100% of CTV in 2016 occurred due to the enhanced dose reduction of V75 Gy compared to the previous years when a high dose was administered to the rectum. In addition, the results shown in Table 4 indicate that the treatment plan at our institution specifies the dose constraint at the maximum dose for PTV excluding the rectum, but does not specify the minimum dose. Therefore, we believe that there are more significant differences between the years for D98% compared to D2%. The scores of the CTV and PTV excluding the rectum changed to a lower degree after 2015 and before 2014 than the OAR scores [Figs. 4(a) and 4(b)]; this may be attributed to the procedure of providing a treatment plan at our institution. In planning prostate IMRT and VMAT, we first select a template that registers the prescribed doses required for optimization and the dose limits required for OAR. Then, the OAR constraints are fine‐tuned as input values while the coverage of the PTV excluding the rectum is prioritized during optimization. We confirmed that the shape of the DVH of the CTV and PTV excluding the rectum remained unchanged during optimization, and the priority was fine‐tuned to reduce the OAR dose. Therefore, the coverage of the PTV excluding the rectum was prioritized in the treatment plan. We believe that for CTV and PTV excluding the rectum, the effect of the difference in the optimization algorithm on the score was small. The degree of improvement in the OAR score significantly differed at higher doses than at lower doses [Figs. 4(c) and 4(d)]. This trend was observed before 2014 and after 2015. Based on the previous treatment results at our institution, it can be concluded that the bladder is associated with fewer side effects than is the rectum. Susil et al.^19^ suggest that the rectum is a dose‐limiting organ in prostate cancer treatment. Therefore, in the PQM scoring table used in this study, the bladder score was set at a value lower than the other scores. Consequently, the bladder had a weaker impact on the overall score than other organs. After 2015, an improving trend was observed in the bladder and rectum scores [Fig. 3(e)]. This result is likely due to the influence of both the difference between PRO2 and PRO3 and the treatment plan’s proficiency. Moreover, we believe that this is due to the fact that the width of the volume criterion relative to the width of the dose distribution point in OAR is narrower in the high‐dose region than in the low‐dose region.

Our findings highlight the value of considering the re‐planning of the optimizing object settings concerning the “difficult region (orange).” Of the 47 patients that underwent re‐planning, more than half (29) underwent treatment plans implemented between 2012 and 2014. The results shown in Figs. 4(c), 4(d), and Table 4 suggest that dose reduction to the OAR was responsible for the increase in the PQM total score after 2015. Moreover until 2014, the TPS version used was 8.9.17 with that version, it was difficult to develop multiple VMAT plans in the time allocated for treatment planning, resulting in the adoption of more IMRT plans. After 2015, the TPS version was upgraded to 11.0.31, which significantly reduced the calculation time for VMAT planning. After the version upgrade, multiple VMAT plans can be developed in the time allocated for treatment planning, thus enabling treatment planning with VMAT alone. Therefore, we believe that the R‐PQM total score was higher than the PQM total score in the present study as the dose to the rectum and bladder could be reduced without compromising the target coverage.

However, the re‐treatment plan used in this study was implemented by a single treatment planner. Therefore, the effect of planner‐related variability cannot be ruled out when more than one planner is involved. It is necessary to share information about the treatment plan with multiple planners before using the treatment planning method obtained in this study in a clinical setting. Furthermore, to minimize the degree of variability in the treatment plan when multiple planners are involved, the use of a knowledge‐based planning tool^20^, ^21^, ^22^ and re‐creation of the treatment plan template based on the empirical results obtained from the re‐planning of this study should be considered.

Finally, in terms of the challenges and prospects of using PlanIQ™, there are currently no clear criteria. It is also up to the user to determine the evaluation results when PlanIQ™ is used as an evaluation tool. However, as discussed, several studies have evaluated treatment plans using PlanIQ™, and we believe that a certain consensus has been reached. In addition, several professional planners have agreed upon the PQM scoring table used in this study, which was ultimately reviewed and approved by radiation oncologists.

The PQM total score calculated using the PQM scoring table showed a strong correlation with the APQM total score. Based on these findings, we believe that the quality of the clinical treatment plan was assured and that it is a validated assessment of the treatment plan. However, the study was limited to a single institution, and the disease site was limited to the prostate. Therefore, additional studies that include multiple sites at multiple institutions are needed. The present study analyzed the largest number of patients in the long term, including the highest proportion of patients in whom PlanIQ™ was employed. As such, we believe our study may provide useful information for use in the performance of clinical research with PlanIQ™.

## 5 CONCLUSIONS

In this study, the APQM total score and PQM total score showed a strong correlation, with an R^2^ of 0.8064. In addition, the PQM total score showed an improving trend in the quality over the course of 8 yr.

Furthermore, 47 patients outside one standard deviation of the ideal PQM total score and APQM total score line were included in the re‐treatment plan. In the re‐treatment planning process, we used the “difficult region (orange)” of the Feasibility DVH^TM^ as a reference point in setting the optimization objectives. All those who underwent re‐treatment planning showed a trend towards improvement, with higher overall scores than those associated with the treatment plan employed for patient delivery in the clinical plan.

In conclusion, the PlanIQ^TM^ provided insights into the quality of the treatment plan at our institution and enabled the identification of problems and areas for improvement in the treatment plan, allowing for the development of appropriate treatment plans for specific patients.

## AUTHOR CONTRIBUTION

The conceptual design of the study was carried out by Motoharu Sasaki, Hitoshi Ikushima, Yuji Nakaguchi and Takeshi Kamomae. Data was collected by Shoji Ueda and Yuto Endo. Data analysis was performed by Satoshi Kobuchi and Kenmei Kuwahara. Interpretation of the submitted papers was discussed by all authors. All authors wrote or critically revised their articles on important intellectual content. In addition, all authors have given final approval to the submitted papers.

## CONFLICT OF INTEREST

Yuji Nakaguchi is an employee of TOYO MEDIC CO., LTD.

## ETHICS REVIEW

This is an observational study that used hospital‐derived data only; we posted a disclosure document rather than an explanatory document or consent form. The posted disclosure document was prepared by the principal investigator and approved by the Tokushima University Hospital clinical research ethics review committee (approval number 3434).
